# p16 protein expression is associated with a poor prognosis in squamous cell carcinoma of the lung

**DOI:** 10.1054/bjoc.1999.0929

**Published:** 2000-01-18

**Authors:** C-I Huang, T Taki, M Higashiyama, N Kohno, M Miyake

**Affiliations:** Department of Thoracic Surgery and Department V of Oncology, Kitano Hospital, Tazuke Kofukai Medical Research Institute, 13-3 Kamiyamacho, Kitaku, Osaka 530-8480, Japan; Department of Surgery, Osaka Medical Center for Cancer and Cardiovascular Diseases, Osaka, Japan; Second Department of Internal Medicine, Ehime University School of Medicine, Ehime, Japan

**Keywords:** p16, lung cancer, prognosis, immunohistochemistry

## Abstract

An immunohistochemical analysis for p16 protein was performed in 171 patients with non-small-cell lung cancer (NSCLC). Sixty-two carcinomas (36.3%) were classified as p16-negative. p16-negative tumours in squamous cell carcinomas (SCCs) were significantly more than those in adenocarcinomas (*P* = 0.039). There was no significant difference in survival according to tumour p16 status in patients with NSCLCs or in patients with adenocarcinomas. In contrast, of patients with SCCs, the 5-year survival rate of patients with p16-negative tumours was significantly lower than those with p16-positive tumours (*P* = 0.001). Especially, the survival of patients with p16-negative tumours was significantly worse than that of patients with p16-positive tumours in the early stage of the SCC, e.g. stage I (*P* = 0.005). Multivariate analysis showed that p16 status and nodal status were significant prognostic factors for the survival of patients with SCCs of the lung (*P* = 0.024 and *P* = 0.008 respectively). In conclusion, our study showed that alteration of p16 was one of the significant factors of a poor prognosis in SCCs of the lung, and that p16 might play an important role in some SCCs of the lung due to its high prevalence and prognostic value. © 2000 Cancer Research Campaign


*British Journal of Cancer* (2000) **82**(2), 374–380
© 2000 Cancer Research Campaign
Article no. bjoc.1999.0929

### p16 protein expression is associated with a poor prognosis in squamous cell carcinoma of the lung

**C-l Huang[1], T Taki[1], M Higashiyama[2], N Kohno[3] and M Miyake[1]**

[1]Department of Thoracic Surgery and Department V of Oncology, Kitano Hospital, Tazuke Kofukai Medical Research Institute, 13-3 Kamiyamacho, Kitaku, Osaka, 530-8480, Japan; [2]Department of Surgery, Osaka Medical Center for Cancer and Cardiovascular Diseases, Osaka, Japan; [3]Second Department of Internal Medicine, Ehime University School of Medicine, Ehime, Japan

**Summary** An immunohistochemical analysis for p16 protein was performed in 171 patients with non-small-cell lung cancer (NSCLC). Sixty-two carcinomas (36.3%) were classified as p16-negative. p16-negative tumours in squamous cell carcinomas (SCCs) were significantly more than those in adenocarcinomas ($P = 0.039$). There was no significant difference in survival according to tumour p16 status in patients with NSCLCs or in patients with adenocarcinomas. In contrast, of patients with SCCs, the 5-year survival rate of patients with p16-negative tumours was significantly lower than those with p16-positive tumours ($P = 0.001$). Especially, the survival of patients with p16-negative tumours was significantly worse than that of patients with p16-positive tumours in the early stage of the SCC, e.g. stage I ($P = 0.005$). Multivariate analysis showed that p16 status and nodal status were significant prognostic factors for the survival of patients with SCCs of the lung ($P = 0.024$ and $P = 0.008$ respectively). In conclusion, our study showed that alteration of p16 was one of the significant factors of a poor prognosis in SCCs of the lung, and that p16 might play an important role in some SCCs of the lung due to its high prevalence and prognostic value. © 2000 Cancer Research Campaign

**Keywords:** p16; lung cancer; prognosis; immunohistochemistry

It is widely accepted that malignant tumours are caused by the accumulation of genetic alterations (Vogelstein et al, 1988; Chung et al, 1995). These genetic alterations regulate the malignant behaviour of solid tumours, such as tumour rapid growth (Cordon-Cardo, 1995) and metastatic potential (Miyake et al, 1991; Dong et al, 1995). Non-small-cell lung cancers (NSCLC) are also variably affected by the activation of oncogenes such as K-*ras* (Rodenhuis and Slebos, 1992), or the inactivation of tumour suppressor genes such as *p53* (Takahashi et al, 1989), *p16*$^{INK4}$ (Vos et al, 1995), and *retinoblastoma (RB)* (Reissmann et al, 1993), which principally control the cell cycle and tumour growth. Such heterogeneity in the aetiologic genetic alterations might be one of the reasons for the variety of clinical behaviours of NSCLCs. Therefore, it is important to classify NSCLCs according to their gene status, which might reflect biological behaviour. Reliable and sensitive prognostic parameters would help us to identify those patients for whom intensive adjuvant therapy is necessary. Especially, it seems justified to evaluate the effects of surgery with adjuvant chemotherapy or radiotherapy.

However, our previous study on mutations of the *p53* and K-*ras* genes in Japanese patients with NSCLC showed that only 41.7% of these patients had mutations in either of the two genes (Huang et al, 1998), and that a genetic classification accounting for only these two genes was insufficient to predict the biological behaviour of NSCLCs. The *p16*$^{INK4A}$ gene belongs to the G1 control gene, which includes *RB* (Betticher et al, 1997). This gene is also believed to be a tumour suppressor gene, and inactivation of *p16*$^{INK4}$ has been detected in various human malignant tumours (Nobori et al, 1994; Reed et al, 1996). Several studies have reported that *p16*$^{INK4}$ alterations occurred in about 27.0–54.0% of NSCLCs (Kratzke et al, 1996; Betticher et al, 1997; Taga et al, 1997; Kashiwabara et al, 1998; Tanaka et al, 1998), but their prognostic significance in NSCLC patients remain unclear (Kratzke et al, 1996; Marchetti et al, 1997; Taga et al, 1997; Volm et al, 1998; Geradts et al, 1999). Therefore, in order to make a more accurate and useful genetic classification of NSCLC, we performed a further study focusing on the *p16*$^{INK4A}$ status.

*Received 3 March 1999*
*Revised 30 June 1999*
*Accepted 7 July 1999*

*Correspondence to:* M Miyake

### MATERIALS AND METHODS

### Clinical characteristics of patients

From January 1991 to December 1994, patients who underwent surgery at the Department of Surgery of the Osaka Medical Center for Cancer and Cardiovascular Diseases and at the Department of Thoracic Surgery of the Kitano Hospital, Medical Research Institute of Osaka in Japan, were studied. Tumour-node-metastasis (TNM) staging designations were made according to the post-surgical pathological international staging system (Mountain, 1997). Because advanced stage lung cancer (stage IV) involved several ill-defined factors and had distant metastases, these patients were excluded from this study. Patients with two or more forms of cancers and those who died of causes other than lung cancer were also excluded from this study. In total, 171 patients with lung cancer up to stage IIIB, which included 106 patients with adenocarcinomas, 55 patients with SCCs and ten patients with large cell carcinomas, were investigated. The patients' clinical records and histopathological diagnoses were fully

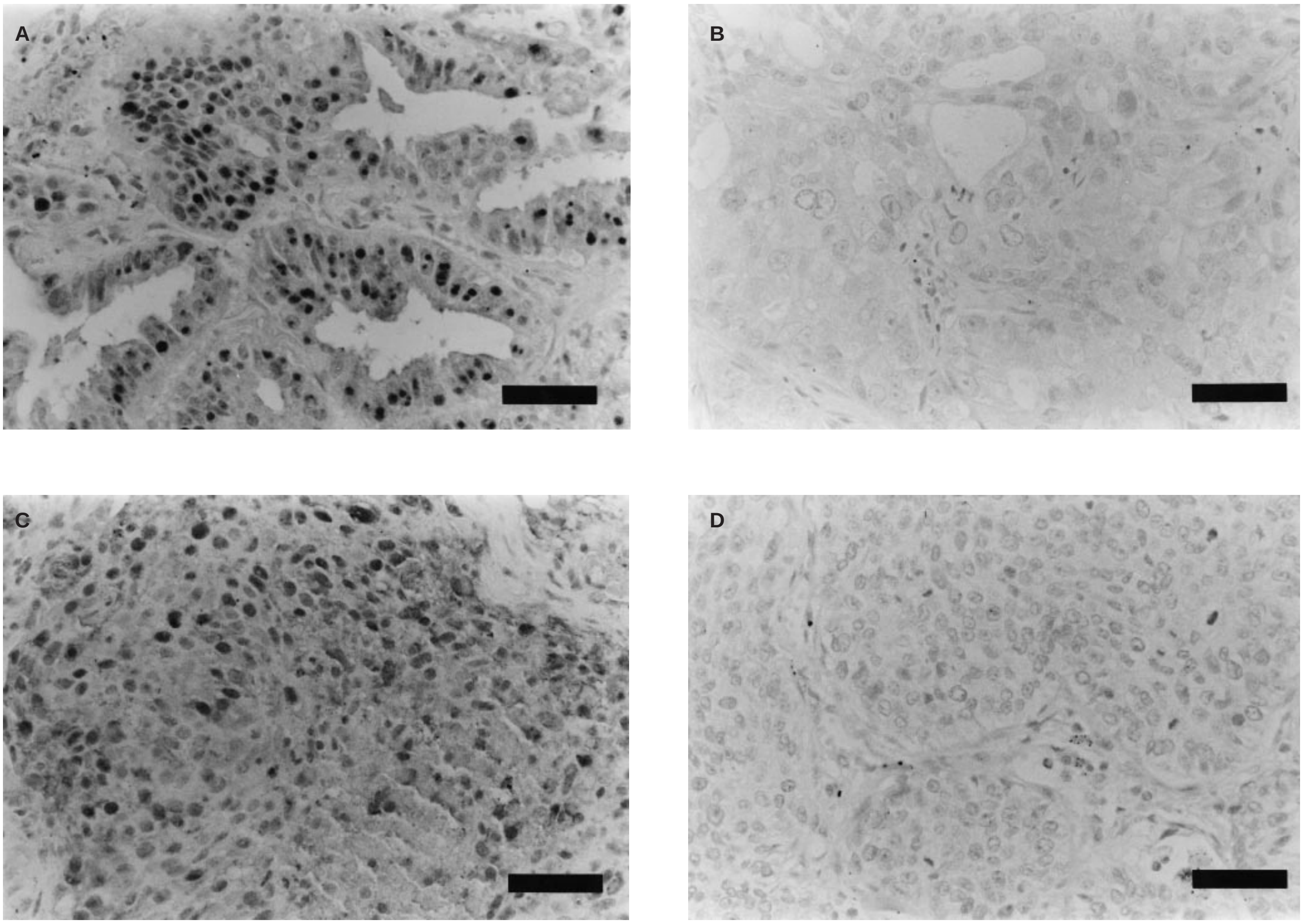

**Figure 1** Immunohistochemical staining of human non-small-cell lung cancer tissues using the avidin–biotin–peroxidase complex procedure (Bars = 50 μm). (**A**) Adenocarcinoma with positive p16 expression. (**B**) Adenocarcinoma with negative p16 expression. (**C**) Squamous cell carcinoma with positive p16 expression. (**D**) Squamous cell carcinoma with negative p16 expression documented. This report includes follow-up data as of 1 March 1998. The median follow-up period for all patients was 58.5 ± 14.3 months.

All patients with N2 or N3 status underwent mediastinal radiotherapy (50 Gy in 25 fractions for a period of 5 weeks) after the surgical resection, and were then treated by two cycles of adjuvant chemotherapy including cisplatin (80 mg per square metre of body-surface area given intravenously (i.v.) on day 1) and vindesine (4 mg per square metre of body-surface area given i.v. on day 1, 8 and 15). Ten patients with T3 or T4 status, who were found to have undergone an incomplete resection, also received radiotherapy (50 Gy in 25 fractions for a period of 5 weeks) for microscopic-positive margins. The other patients had no radiotherapy before recurrence. Post-operative adjuvant systemic chemotherapy was given according to the nodal status. Of the 69 node-positive patients, 63 patients underwent adjuvant chemotherapy with two cycles of cisplatin and vindesine. Of the 102 node-negative patients, 97 patients did not have any further adjuvant treatments. We detected distant metastases in 75 patients during the observation period, and among these 75 patients, 11 patients also had local recurrences. Six patients had only locoregional recurrences. After the recurrence, the locoregional tumours or lymph nodes were principally treated with radiotherapy. Patients with distant metastases were treated with a different chemotherapy regime including cisplatin and etoposide.

### Immunohistochemistry for p16 protein expression

We studied the immunohistochemical study for p16 protein expression using a mouse monoclonal antibody detecting the full-length human p16 protein (PharMingen, Inc., San Diego, CA, USA, clone G175–405) as described previously (Betticher et al, 1997; Vonlanthen et al, 1998). In a pilot study, initially we performed the immunohistochemistry with microwave pretreatment and that without microwave pretreatment. Thus, microwave pretreatment was preferable because of good contrast using this antibody. Formalin-fixed paraffin-embedded tissues were cut in 4-μm sections and mounted on poly-L-lysine-coated slides. The sections were deparaffinized and rehydrated. The slides were heated in a microwave for 10 min in a 10-mmol $l^{-1}$ citrate buffer solution at pH 6.0, and cooled down to room temperature for at least 20 min. After quenching the endogenous peroxidase activity with 0.3% hydrogen peroxide (in absolute methanol) for 30 min, the sections were treated for 2 h at room temperature with 5% bovine serum albumin to block non-specific staining. Subsequently, the slides were incubated at 4°C overnight with the

*© 2000 Cancer Research Campaign*

**Table 1** Distribution of 171 non-small-cell lung cancer patients according to p16 status

| Histology variables | Non-small-cell lung cancer *n* | p16+ | p16– | *P*-value | Adenocarcinoma *n* | p16+ | p16– | *P*-value | Squamous cell carcinoma *n* | p16+ | p16– | *P*-value |
|---|---|---|---|---|---|---|---|---|---|---|---|---|
| Smoking | | | | | | | | | | | | |
| Non-smoker | 49 | 34 | 15 | 0.331 | 44 | 31 | 13 | 0.638 | 4 | 2 | 2 | 0.910 |
| Smoker | 122 | 75 | 47 | | 62 | 41 | 21 | | 51 | 27 | 24 | |
| Age at surgery | | | | | | | | | | | | |
| ≤60 | 60 | 41 | 19 | 0.359 | 44 | 33 | 11 | 0.189 | 12 | 5 | 7 | 0.385 |
| >60 | 111 | 68 | 43 | | 62 | 39 | 23 | | 43 | 24 | 19 | |
| Sex | | | | | | | | | | | | |
| Male | 127 | 80 | 47 | 0.729 | 68 | 46 | 22 | 0.935 | 50 | 27 | 23 | 0.659 |
| Female | 44 | 29 | 15 | | 38 | 26 | 12 | | 5 | 2 | 3 | |
| Tumour status | | | | | | | | | | | | |
| T1 | 46 | 33 | 13 | 0.453 | 30 | 23 | 7 | 0.527 | 14 | 8 | 6 | 0.464 |
| T2 | 77 | 45 | 32 | | 45 | 30 | 15 | | 26 | 11 | 15 | |
| T3 | 34 | 21 | 13 | | 21 | 12 | 9 | | 11 | 7 | 4 | |
| T4 | 14 | 10 | 4 | | 10 | 7 | 3 | | 4 | 3 | 1 | |
| Nodal status | | | | | | | | | | | | |
| N0 | 102 | 66 | 36 | 0.363 | 61 | 40 | 21 | 0.168 | 35 | 22 | 13 | 0.207 |
| N1 | 24 | 13 | 11 | | 12 | 7 | 5 | | 10 | 4 | 6 | |
| N2 | 38 | 27 | 11 | | 28 | 23 | 5 | | 9 | 3 | 6 | |
| N3 | 7 | 3 | 4 | | 5 | 2 | 3 | | 1 | 0 | 1 | |
| Pathological stage | | | | | | | | | | | | |
| Stage I | 80 | 51 | 29 | 0.981 | 47 | 33 | 14 | 0.270 | 29 | 16 | 13 | 0.428 |
| Stage II | 36 | 22 | 14 | | 21 | 11 | 10 | | 11 | 7 | 4 | |
| Stage IIIA | 35 | 23 | 12 | | 24 | 19 | 5 | | 10 | 3 | 7 | |
| Stage IIIB | 20 | 13 | 7 | | 14 | 9 | 5 | | 5 | 3 | 2 | |
| Differentiation | | | | | | | | | | | | |
| Well | 33 | 22 | 11 | 0.319 | 26 | 20 | 6 | 0.444 | 7 | 2 | 5 | 0.243 |
| Moderately | 81 | 47 | 34 | | 48 | 30 | 18 | | 33 | 17 | 16 | |
| Poorly | 57 | 40 | 17 | | 32 | 22 | 10 | | 15 | 10 | 5 | |
| Histology | | | | | | | | | | | | |
| Adeonocarcinoma | 106 | 72 | 34 | 0.039 | | | | | | | | |
| Squamous cell carcinoma | 55 | 29 | 26 | | | | | | | | | |
| Large cell carcinoma | 10 | 8 | 2 | | | | | | | | | |
| Total number of patients | 171 | 109 | 62 | | 106 | 72 | 34 | | 55 | 29 | 26 | |

anti-p16 antibody at a 1:100 dilution. Then, the slides were washed three times in phosphate-buffered saline (PBS), and then incubated for 1 h with biotinylated horse anti-mouse IgG. After being washed three times in PBS, the sections were incubated with the avidin–biotin–peroxidase complex (Vector) for 1 h and then washed in PBS for 10 min. Antibody binding was visualized with 3,3′-diaminobenzidine tetrahydrochloride, and the sections counterstained lightly with Mayer's haematoxylin. There was no difference in staining intensity between peripheral and central portions of the tissues, except for the edge effect, common to immunohistochemical assays. All immunostained sections were reviewed by two pathologists who had no knowledge of the patients' clinical status.

p16 immunostaining was assessed by comparison of type II pneumocytes, normal bronchial cells and some endothelial cells as the positive internal control. And, small lymphocytes without nuclear staining of p16 were used as a negative internal control. For scoring the p16 staining patterns, only nuclear staining above any cytoplasmic background was considered to be positive for p16 protein expression. Tumours were classified as p16-positive (p16+) when the proportion of stained nuclei was ≥ 10% of all nuclei in the tumour. Thus, tumours were evaluated as p16-negative (p16–) when the proportion of stained nuclei was < 10% of all nuclei in the tumour. Cytoplasmic staining alone was considered as p16-negative (Taga et al, 1997; Gazzeri et al, 1998).

### Statistical analysis

Clinical data including smoking habits were extracted from the clinical records. The overall cancer-specific survival was defined from the data of the operation to the data of cancer death. The statistical significance of differences in p16 status in relation to several clinical and pathological parameters was assessed by the $\chi^2$ test. The Kaplan–Meier method was used to estimate the probability of overall survival as function of time, and the survival periods were compared using the log-rank test (Kaplan and Meier, 1958; Mantel, 1966). Multivariate analyses were performed using the Cox regression model with the SAS statistical package (SAS Institute, Cary, NC, USA) (Cox, 1972). Scores were assigned to each variable for the regression analysis. All *P*-values were based on two-tailed statistical analysis, and a *P*-value of less than 0.05 was considered to indicate statistical significance.

### RESULTS

### Distribution of p16 protein expression in resected NSCLC tumours

Of the 171 NSCLCs we studied, 23 carcinomas (13.5%) had nuclear staining in ≥ 70% of tumour cells, 45 carcinomas (26.3%) had nuclear staining in 30–70% of tumour cells, 41 carcinomas

 *© 2000 Cancer Research Campaign*

**Table 2** Five-year survival rate of 171 non-small-cell lung cancer patients according to p16 status

| Histology variables | Non-small-cell lung cancer p16+ | p16– | *P*-value | Adenocarcinoma p16+ | p16– | *P*-value | Squamous cell carcinoma p16+ | p16– | *P*-value |
|---|---|---|---|---|---|---|---|---|---|
| Smoking | | | | | | | | | |
| Non-smoker | 71.9 | 65.2 | 0.353 | 72.5 | 75.2 | 0.769 | 100.0 | 0.0 | 0.333 |
| Smoker | 58.5 | 46.4 | 0.179 | 41.5 | 34.9 | 0.985 | 85.2 | 49.2 | 0.006 |
| Age at surgery | | | | | | | | | |
| ≤60 | 50.9 | 44.4 | 0.807 | 48.6 | 29.1 | 0.866 | 80.0 | 38.1 | 0.299 |
| >60 | 69.1 | 52.7 | 0.028 | 59.0 | 54.8 | 0.721 | 87.5 | 47.4 | 0.003 |
| Sex | | | | | | | | | |
| Male | 60.9 | 46.9 | 0.119 | 47.5 | 39.0 | 0.766 | 85.2 | 47.4 | 0.004 |
| Female | 67.2 | 64.2 | 0.441 | 67.5 | 72.9 | 0.860 | 100.0 | 33.3 | 0.400 |
| Tumour status | | | | | | | | | |
| T1 | 87.9 | 52.7 | 0.008 | 87.0 | 57.1 | 0.065 | 100.0 | 44.4 | 0.046 |
| T2 | 59.2 | 53.2 | 0.298 | 49.8 | 52.4 | 0.783 | 90.9 | 46.7 | 0.019 |
| T3 | 42.9 | 46.2 | 0.645 | 25.0 | 55.6 | 0.192 | 71.4 | 25.0 | 0.293 |
| T4 | 30.0 | 0.0 | 0.351 | 14.3 | 0.0 | 0.225 | 66.7 | 0.0 | >0.999 |
| Nodal status | | | | | | | | | |
| N0 | 83.2 | 60.7 | 0.003 | 77.5 | 62.3 | 0.193 | 95.5 | 51.3 | <0.001 |
| N1 | 44.9 | 63.6 | 0.665 | 28.6 | 80.0 | 0.179 | 75.0 | 50.0 | 0.435 |
| N2 | 22.2 | 27.3 | 0.783 | 17.4 | 20.0 | 0.217 | 33.3 | 33.3 | 0.749 |
| N3 | 0.0 | 0.0 | 0.153 | 0.0 | 0.0 | 0.207 | (–) | 0.0 | ND |
| Pathological stage | | | | | | | | | |
| Stage I | 90.0 | 58.6 | <0.001 | 84.8 | 58.9 | 0.104 | 100.0 | 51.3 | 0.005 |
| Stage II | 54.2 | 71.4 | 0.502 | 36.4 | 80.0 | 0.098 | 85.7 | 50.0 | 0.155 |
| Stage IIIA | 26.1 | 25.0 | 0.520 | 42.1 | 20.0 | 0.105 | 33.3 | 28.6 | 0.772 |
| Stage IIIB | 23.1 | 0.0 | 0.578 | 0.0 | 0.0 | 0.281 | 66.7 | 0.0 | 0.592 |
| Differentiation | | | | | | | | | |
| Well | 81.8 | 63.6 | 0.227 | 80.0 | 66.7 | 0.403 | 100.0 | 60.0 | >0.999 |
| Moderately | 63.0 | 52.5 | 0.361 | 51.9 | 60.5 | 0.508 | 82.4 | 41.7 | 0.031 |
| Poorly | 50.8 | 40.3 | 0.292 | 34.1 | 30.0 | 0.803 | 90.0 | 0.0 | 0.025 |
| Total number of patients | 62.2 | 51.0 | 0.071 | 54.3 | 51.8 | 0.768 | 86.2 | 45.5 | 0.001 |

ND, not done.

(24.0%) had nuclear staining in 10–30% of tumour cells, and 62 carcinomas (36.3%) had nuclear staining in <10% of tumour cells. Thus, 109 carcinomas (63.7%) were classified as p16-positive, and 62 carcinomas (36.3%) were p16-negative (Figure 1 and Table 1). Of the 106 adenocarcinomas of the lung, 34 tumours (32.1%) were p16-negative. Of the 55 SCCs, 26 tumours (47.3%) were p16-negative. p16-negative tumours in SCCs were significantly more than those in adenocarcinomas ($P = 0.039$). However, there were no statistically significant relationships between p16 expression and smoking habits, the patient's age at surgery, gender, tumour status, nodal status, pathological stage and tumour differentiation (Table 1).

### Overall survival of patients with NSCLCs in relation to p16 status

The 5-year survival rate of all 171 patients stratified according to their p16 status was shown in Table 2. No significant difference was found in the 5-year survival of NSCLC patients with p16-negative tumours versus those with p16-positive tumours (51.0% vs 62.2%, Figure 2A and Table 2). However, the survival of patients with p16-negative tumours was significantly worse than that of patients with p16-positive tumours in the early stage of NSCLC, such as stage I, T1 status and N0 status ($P < 0.001$, $P = 0.008$ and $P = 0.003$ respectively; Figure 2B and Table 2).

A Cox regression analysis was performed to evaluate prognostic factors for NSCLC, as shown in Table 3. Among the 171 NSCLC patients, only the nodal status was found to be a significant factor for the prognosis (hazard ratio = 1.956, $P < 0.001$).

### Overall survival of patients with adenocarcinomas of the lung in relation to p16 status

The 5-year survival rate of the 106 patients with adenocarcinomas stratified according to their p16 status is shown in Table 2. There was no significant difference in the 5-year survival between patients with p16-negative adenocarcinomas and those with p16-positive adenocarcinomas (51.8% vs 54.3%, Figure 2C), even in the early stage of adenocarcinoma.

A Cox regression analysis to evaluate prognostic factors for adenocarcinoma showed that two variables, the tumour status (hazard ratio = 1.488, $P = 0.024$) and the nodal status (hazard ratio = 1.756, $P < 0.001$) were found to be significant factors for the prognosis (Table 3).

### Overall survival of patients with SCCs of the lung in relation to p16 status

Of the 55 patients with squamous cell carcinomas, the 5-year survival rate of patients with p16-negative tumours was significantly lower than those with p16-positive tumours (45.5% vs 86.2%, $P = 0.001$, Figure 2D and Table 2). Especially, the survival of patients with p16-negative tumours was significantly worse than those with p16-positive tumours in the early stage of SCC, such as stage I, T1 status and N0 status ($P = 0.005$, $P = 0.046$ and $P < 0.001$ respectively; Figure 2E and Table 2). In addition, the survival of patients with p16-negative tumours was significantly worse than those with p16-positive tumours in moderately and poorly differentiated tumours ($P = 0.031$ and $P = 0.025$ respectively).

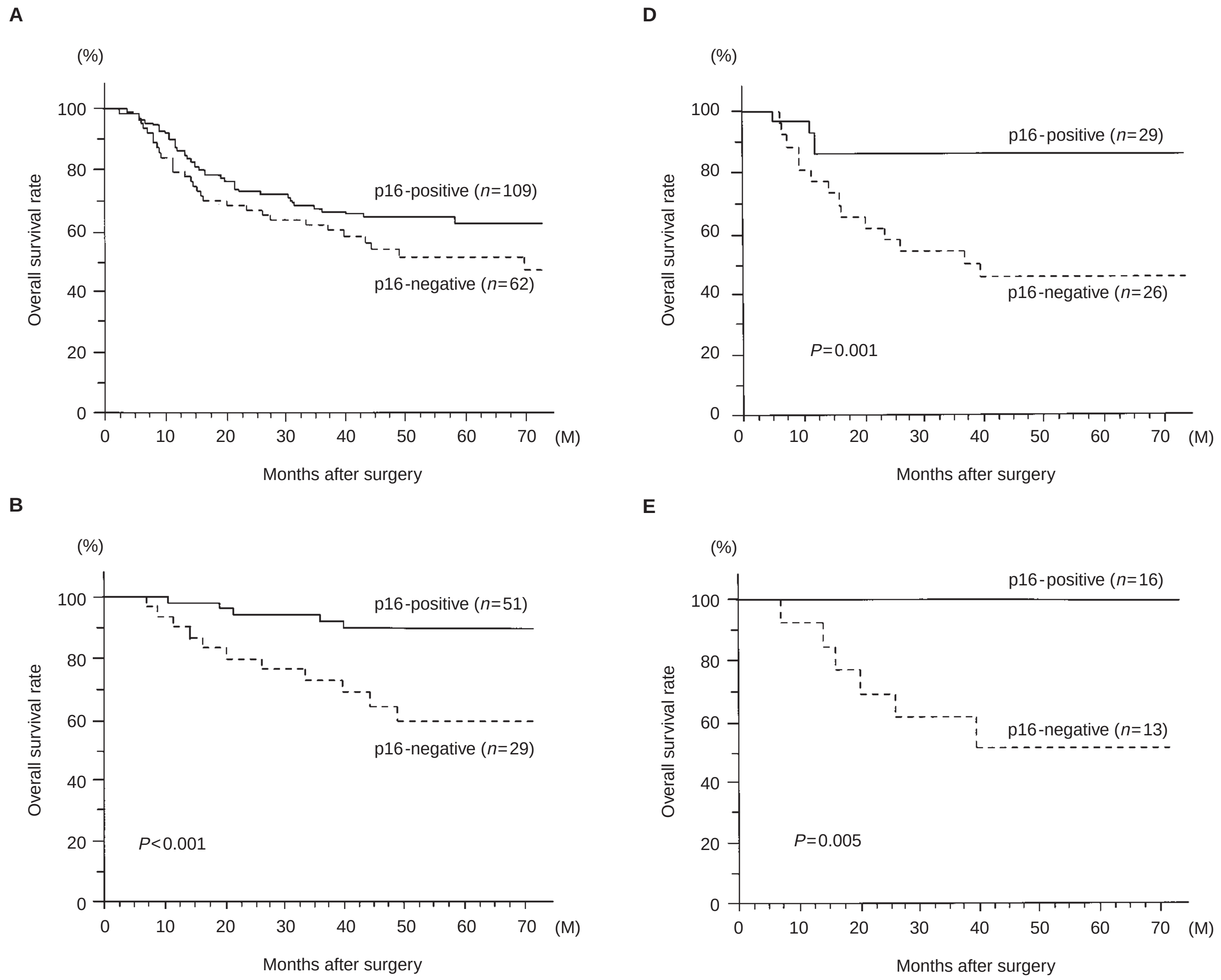

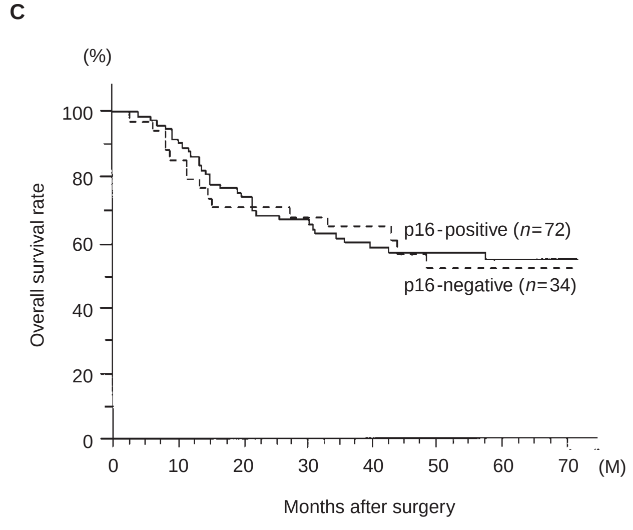

**Figure 2** (**A**) Overall survival of 171 patients with non-small-cell lung cancers according to their tumour p16 status. (**B**) Overall survival of 80 patients with stage I NSCLCs according to their tumour p16 status. (**C**) Overall survival of 106 patients with adenocarcinomas of the lung according to their tumour p16 status. (**D**) Overall survival of 55 patients with squamous cell carcinomas of the lung according to their tumour p16 status. (**E**) Overall survival of 29 patients with stage I squamous cell carcinomas of the lung according to their tumour p16 status

A Cox regression analysis to evaluate the prognostic factors for SCC showed that two variables, the p16 status (hazard ratio = 3.939, $P = 0.024$) and the nodal status (hazard ratio = 2.310, $P = 0.008$) were significant factors for the prognosis (Table 3).

### DISCUSSION

*p16^INK4A^* is located at 9p21 on chromosome and encodes a cyclin-dependent kinase (cdk) inhibitor which is an important cell cycle regulatory element (Serrano et al, 1993). The p16 protein binds both cdk4 and cdk6, and negatively regulates the cyclin D-dependent phosphorylation of the RB protein, thereby inhibiting the cell cycle transition from G1 to S phase by sequestering of a transactivation factor of E2F (Serrano et al, 1993; Lukas et al, 1995). Therefore, a loss of p16 function could disrupt the RB-mediated tumour suppressor pathway. The mechanisms of inactivation of *p16^INK4A^* in patients with NSCLCs are believed to be a homozygous deletion (Shimizu and Sekiya, 1995; Vos et al, 1995) and hypermethylation of the CpG island in the promoter region of the *p16^INK4A^* gene (Herman et al, 1995; Merlo et al, 1995). In addition, mutations of the *p16^INK4A^* gene were not detected frequently in

*© 2000 Cancer Research Campaign*

**Table 3** Multivariate regression analysis in predicting survival of 171 patients with non-small-cell lung cancer

| Histology | | Non-small-cell lung cancer | | | Adenocarcinoma | | | Squamous cell carcinoma | | |
|---|---|---|---|---|---|---|---|---|---|---|
| variables | Assigned score | Hazard ratio | (95% CI) | *P*-value | Hazard ratio | (95% CI) | *P*-value | Hazard ratio | (95% CI) | *P*-value |
| p16 status | | | | | | | | | | |
| Positive | 0 | 1.225 | (0.749–2.003) | 0.419 | 0.764 | (0.401–1.455) | 0.413 | 3.939 | (1.196–12.972) | 0.024 |
| Negative | 1 | | | | | | | | | |
| Smoking | | | | | | | | | | |
| Non-smoker | 0 | 1.235 | (0.517–2.954) | 0.635 | 1.613 | (0.568–4.581) | 0.369 | 0.465 | (0.090–2.404) | 0.361 |
| Smoker | 1 | | | | | | | | | |
| Age at surgery | | | | | | | | | | |
| <60 | 0 | 0.992 | (0.601–1.637) | 0.975 | 1.200 | (0.642–2.243) | 0.568 | 1.418 | (0.472–4.262) | 0.534 |
| ≥60 | 1 | | | | | | | | | |
| Sex | | | | | | | | | | |
| Male | 0 | 0.782 | (0.336–1.819) | 0.568 | 0.682 | (0.255–1.825) | 0.446 | 1.900 | (0.363–9.938) | 0.447 |
| Female | 1 | | | | | | | | | |
| Tumour status | | | | | | | | | | |
| T1 | 1 | 1.250 | (0.958–1.633) | 0.101 | 1.488 | (1.053–2.101) | 0.024 | 0.921 | (0.490–1.730) | 0.798 |
| T2 | 2 | | | | | | | | | |
| T3 | 3 | | | | | | | | | |
| T4 | 4 | | | | | | | | | |
| Nodal status | | | | | | | | | | |
| N0 | 0 | 1.956 | (1.533–2.496) | <0.001 | 1.756 | (1.316–2.343) | <0.001 | 2.310 | (1.248–4.276) | 0.008 |
| N1 | 1 | | | | | | | | | |
| N2 | 2 | | | | | | | | | |
| N3 | 3 | | | | | | | | | |
| Differentiation | | | | | | | | | | |
| Well | 0 | 1.313 | (0.958–1.633) | 0.142 | 1.413 | (0.907–2.203) | 0.127 | 1.202 | (0.589–2.453) | 0.613 |
| Moderately | 1 | | | | | | | | | |
| Poorly | 2 | | | | | | | | | |

patients with NSCLCs (Nakagawa et al, 1995; Gazzeri et al, 1998). The clinical significance of the p16 status in NSCLCs is still controversial, partly because various methods have been used in a relatively small population of patients. Recently, the immunohistochemical detection of p16 is considered to be a sensitive and suitable method to screen for p16 alterations and appears to be superior to any other method for detecting genetic alterations (Gazzeri et al, 1998). Therefore, we evaluated the expression of the p16 protein by immunohistochemistry in this study.

Of the 171 NSCLCs we studied, 23 carcinomas (13.5%) showed strong p16 protein expression with nuclear staining in > 70% of tumour cells. Such tumours might be associated with an inactive RB protein. The $p16^{INK4}$ and *RB* genes constitute a single pathway and regulate each other by a feedback mechanism. Several previous studies have reported the inverse relationship between $p16^{INK4}$ expression and *RB* expression (Otterson et al, 1994; Shapiro et al, 1995; Sakaguchi et al, 1996).

As previously reported, there was no relationship between the p16 status and the pathological stage in adenocarcinomas as well as in SCCs. Twenty-nine (36.3%) of 80 patients with stage I NSCLCs had alterations of p16. Therefore, loss of p16 occurs at a relatively early stage, and may play an important role in the progression of some NSCLCs (Kinoshita et al, 1996; Taga et al, 1997). Previous clinical studies have reported that genetic alterations of $p16^{INK4}$ occurred in about 27.0–54.0% of resected NSCLCs (Kratzke et al, 1996; Betticher et al, 1997; Taga et al, 1997; Kashiwabara et al, 1998; Tanaka et al, 1998), which is in agreement with our results. Furthermore, with respect to the histology of NSCLC, p16 alterations have been reported to occur in 21.2–75.0% of SCCs, and in 20.3–41.5% of adenocarcinomas. Most studies have shown a high prevalence of p16 alterations in SCCs, as our results confirmed.

With respect to the survival of NSCLC patients, our study demonstrated that the alteration of p16 was one of the significant factors for a poor prognosis in SCCs of the lung, especially in the early stage such as stage I and N0 status. Several studies also reported that the loss of p16 was one of the significant factors for the poor survival in NSCLCs, especially in early stage of SCC (Kratzke et al, 1996; Taga et al, 1997). On the Cox multivariate analysis of SCCs, the p16 status had a hazard ratio of 3.939 for survival. The 5-year survival of patients with p16-negative SCC was only 51.3% even in stage I. On the other hand, no patient with stage I SCC had a recurrence after the surgery in our series. Therefore, in patients with p16-negative SCCs of the lung, adjuvant chemoradiotherapy after surgery might be useful even if they are in the early stage, whereas no adjuvant therapy might be recommended for surgically-treated patients with p16-positive SCC in the early stage.

However, the loss of p16 expression had no prognostic impact in adenocarcinomas of the lung, which coincided with the previous report (Volm et al, 1998). From previous clinical studies including ours, mutations of *p53* and mutations of K-*ras* have been reported to be useful factors indicative of a poor prognosis in adenocarcinomas of the lung (Fukuyama et al, 1997; Huang et al, 1998). The different biological mechanisms among $p16^{INK4}$, *p53* and K-*ras* might have a strong effect on the survival between patients with SCCs and those with adenocarcinomas. For example, with respect to the clinical behaviour of the tumour, SCCs are likely to relapse in locoregional region, whereas adenocarcinomas are likely to have distant metastasis even when the primary tumours are still small (Minna, 1991). The alterations of $p16^{INK4}$ gene have been reported to be associated with tumour proliferative activity and tumour growth. On the other hand, mutations of the K-*ras* gene, which was believed to be specific for adenocarcinomas of the

© 2000 Cancer Research Campaign lung, were considered to regulate not only cell proliferation but also angiogenesis via vascular endothelial growth factor (Rak et al, 1995). Since angiogenesis is essential for tumour growth and distant metastasis, mutations of the K-*ras* gene will induce tumour aggressiveness (Folkman, 1990).

In conclusion, our study showed that alterations of p16 was one of the significant factor for a poor prognosis in SCCs, and might play a specific and important role in SCCs of the lung because of its high prevalence and significant prognostic value.

### REFERENCES

Betticher DC, White GRM, Vonlanthen S, Liu X, Kappeler A, Altermatt HJ, Thatcher N and Heighway J (1997) G1 control gene status is frequently altered in resectable non-small cell lung cancer. *Int J Cancer* **74**: 556–562

Chung GTY, Sundaresan V, Hasleton P, Rudd R, Taylor R and Rabbitts PH (1995) Sequential molecular genetic changes in lung cancer development. *Oncogene* **11**: 2591–2598

Cordon-Cardo C (1995) Mutation of cell cycle regulators: biological and clinical implications for human neoplasia. *Am J Pathol* **147**: 545–560

Cox DR (1972) Regression models and life-tables. *J R Stat Soc B* **34**: 187–220

Dong JT, Lamb PW, Rinker-Schaeffer CW, Vukanovic J, Ichikawa T, Isaacs JT and Barrett JC (1995) KAI1, a metastasis suppressor gene for prostate cancer on human chromosome 11p11.2. *Science* **268**: 884–886

Folkman J (1990) What is the evidence that tumors are angiogenesis dependent? *J Natl Cancer Inst* **82**: 4–6

Fukuyama Y, Mitsudomi T, Sugio K, Ishida T, Akazawa K and Sugimachi K (1997) K-ras and p53 mutations are an independent unfavourable prognostic indicator in patients with non-small-cell lung cancer. *Br J Cancer* **75**: 1125–1130

Gazzeri S, Gouyer V, Vour'ch C, Brambilla C and Brambilla E (1998) Mechanisms of $p16^{INK4A}$ inactivation in non-small-cell lung cancers. *Oncogene* **16**: 497–504

Geradts J, Fong KM, Zimmerman PV, Maynard R and Minna JD (1999) Correlation of abnormal RB, $p16^{ink4a}$, and p53 expression with 3p loss of heterozygosity, other genetic abnormalities, and clinical features in 103 primary non-small-cell lung cancers. *Clin Cancer Res* **5**: 791–800

Herman JG, Merlo A, Mao L, Lapidus RG, Issa JPJ, Davidson NE, Sidransky D and Baylin SB (1995) Inactivation of the CDKN2/p16/MTS1 gene is frequently associated with aberrant DNA methylation in all common human cancers. *Cancer Res* **55**: 4525–4530

Huang C, Taki T, Adachi M, Konishi T, Higashiyama M, Kinoshita M, Hadama T and Miyake M (1998) Mutations of *p53* and K-*ras* genes as prognostic factors for non-small-cell lung cancer. *Int J Oncol* **12**: 553–563

Kaplan EL and Meier P (1958) Non-parametric estimation from incomplete observations. *J Am Stat Assoc* **53**: 457–481

Kashiwabara K, Oyama T, Sano T, Fukuda T and Nakajima T (1998) Correlation between methylation status of the *p16/CDKN2* gene and the expression of p16 and Rb proteins in primary non-small-cell lung cancers. *Int J Cancer* **79**: 215–220

Kawamata N, Miller CW and Koeffler HP (1995) Molecular analysis of a family of cyclin-dependent kinase inhibitor genes (*p15/MTS2/INK4b* and *p18/INK4c*) in non-small-cell lung cancers. *Mol Carcinogen* **14**: 263–268

Kinoshita I, Dosaka-Akita H, Mishina T, Akie K, Nishi M, Hiroumi H, Hommura F and Kawakami Y (1996) Altered $p16^{INK4}$ and retinoblastoma protein status in non-small-cell lung cancer: potential synergistic effect with altered p53 protein on proliferative activity. *Cancer Res* **56**: 5557–5562

Kratzke RA, Greatens TM, Rubins JB, Maddaus MA, Niewoehner DE, Niehans GA and Geradts J (1996) Rb and $p16^{INK4a}$ expression in resected non-small-cell lung tumors. *Cancer Res* **56**: 3415–3420

Lukas J, Parry D, Aagaard L, Mann DJ, Bartkova J, Strauss M, Peters G and Bartek J (1995) Retinoblastoma-protein-dependent cell-cycle inhibition by the tumour suppressor p16. *Nature* **375**: 503–506

Mantel N (1966) Evaluation of survival data and two new rank order statistics arising in its consideration. *Cancer Chemother Rep* **50**: 163–170

Marchetti A, Buttitta F, Pellegrini S, Bertacca G, Chella A, Carnicelli V, Tognoni V, Filardo A, Angeletti CA and Bevilacqua G (1997) Alterations of p16 (MTS1) in node-positive non-small-cell lung carcinomas. *J Pathol* **181**: 178–182

Merlo A, Herman JG, Mao L, Lee DJ, Gabrielson E, Burger PC, Baylin SB and Sidransky D (1995) 5′ CpG island methylation is associated with transcriptional silencing of the tumour suppressor p16/CDKN2/MTS1 in human cancers. *Nat Med* **1**: 686–692

Minna JD (1991) Neoplasms of the lung. In: *Principles of Internal Medicine*, Isselbacher KJ (ed), pp. 1102–1110. McGraw-Hill: New York

Miyake M, Koyama M, Seno M and Ikeyama S (1991) Identification of the motility-related protein (MRP-1), recognized by monoclonal antibody M31-15, which inhibits cell motility. *J Exp Med* **174**: 1347–1354

Mountain CF (1997) Revisions in the international system for staging lung cancer. *Chest* **111**: 1710–1717

Nakagawa K, Conrad NK, Williams JP, Johnson BE and Kelley MJ (1995) Mechanism of inactivation of CDKN2 and MTS2 in non-small-cell lung cancer and associated with advanced stage. *Oncogene* **11**: 1843–1851

Nobori T, Miura K, Wu DJ, Lois A, Takabayashi K and Carson DA (1994) Deletions of the cyclin-dependent kinase-4 inhibitor gene in multiple human cancers. *Nature* **368**: 753–756

Otterson GA, Kratzke RA, Coxon A, Kim YW and Kaye FJ (1994) Absence of $p16^{INK4}$ protein is restricted to the subset of lung cancer lines that retains wildtype RB. *Oncogene* **9**: 3375–3378

Rak J, Mitsuhashi Y, Bayko L, Filmus J, Shirasawa S, Sasazuki T and Kerbel RS (1995) Mutant ras oncogenes up-regulate VEGF/VPF expression: implications for induction and inhibition of tumor angiogenesis. *Cancer Res* **55**: 4575–4580

Reed AL, Califano J, Cairns P, Westra WH, Jones RM, Koch W, Ahrendt S, Eby Y, Sewell D, Nawroz H, Bartek J and Sidransky D (1996) High frequency of p16 (CDKN2/MTS-1/INK4A) inactivation in head and neck squamous cell carcinoma. *Cancer Res* **56**: 3630–3633

Reissmann PT, Koga H, Takahashi R, Figlin RA, Holmes EC, Piantadosi S, Cordon-Cardo C and Slamon DJ (1993) Inactivation of the retinoblastoma susceptibility gene in non-small-cell lung cancer. The lung cancer study group. *Oncogene* **8**: 1913–1919

Rodenhuis S and Slebos RJC (1992) Clinical significance of ras oncogene activation in human lung cancer. *Cancer Res* **52**: 2665s–2669s

Rusin MR, Okamoto A, Chorazy M, Czyzewski K, Harasim J, Spillare EA, Hagiwara K, Hussain SP, Xiong Y, Demetrick DJ and Harris CC (1996) Intragenic mutations of the $p16^{INK4}$, $p15^{INK4B}$ and p18 genes in primary non-small-cell lung cancers. *Int J Cancer* **65**: 734–739

Sakaguchi M, Fujii Y, Hirabayashi H, Yoon HE, Komoto Y, Oue T, Kusafuka T, Okada A and Matsuda H (1996) Inversely correlated expression of p16 and Rb protein in non-small cell lung cancers: an immunohistochemical study. *Int J Cancer* **65**: 442–445

Serrano M, Hannon GJ and Beach D (1993) A new regulatory motif in cell-cycle control causing specific inhibition of cyclin D/CDK4. *Nature* **366**: 704–707

Shapiro GI, Edwards CD, Kobzik L, Godleski J, Richards W, Sugarbaker DJ and Rollins BJ (1995) Reciprocal Rb inactivation and $p16^{INK4}$ expression in primary lung cancers and cell lines. *Cancer Res* **55**: 505–509

Shimizu T and Sekiya T (1995) Loss of heterozygosity at 9p21 loci and mutations of the *MTS1* and *MTS2* genes in human lung cancers. *Int J Cancer* **63**: 616–620

Taga S, Osaki T, Ohgami A, Imoto H, Yoshimatsu T, Yoshino I, Yano K, Nakanishi R, Ichiyoshi Y and Yasumoto K (1997) Prognostic value of the immunohistochemical detection of $p16^{INK4}$ expression in non-small cell lung carcinoma. *Cancer* **80**: 389–395

Takahashi T, Nau MM, Chiba I, Birrer MJ, Rosenberg RK, Vinocour M, Levitt M, Pass H, Gazdar AF and Minna JD (1989) p53: a frequent target for genetic abnormalities in lung cancer. *Science* **246**: 491–494

Tanaka H, Fujii Y, Hirabayashi H, Miyoshi S, Sakaguchi M, Yoon HE and Matsuda H (1998) Disruption of the RB pathway and cell-proliferative activity in non-small-cell lung cancers. *Int J Cancer* **79**: 111–115

Vogelstein B, Fearon ER, Hamilton SR, Kern SE, Preisinger AC, Leppert M, Nakamura Y, White R, Smits AMM and Bos JL (1988) Genetic alterations during colorectal-tumor development. *N Engl J Med* **319**: 525–532

Volm M, Koomagi R and Mattern J (1998) Prognostic value of $p16^{INK4A}$ expression in lung adenocarcinoma. *Anticancer Res* **18**: 2309–2312

Vonlanthen S, Heighway J, Tschan MP, Borner MM, Altermatt HJ, Kappeler A, Tobler A, Fey MF, Thatcher N, Yarbrough WG and Betticher DC (1998) Expression of p16INK4a/p16α and p19ARF/p16β is frequently altered in non-small-cell lung cancer and correlates with p53 overexpression. *Oncogene* **17**: 2779–2785

Vos S, Miller CW, Takeuchi S, Gombart AF, Cho SK and Koeffler HP (1995) Alterations of CDKN2 (p16) in non-small-cell lung cancer. *Gene Chromosome Canc* **14**: 164–170

 © 2000 Cancer Research Campaign
